# Supplementary material for: Cardiometabolic Risk Markers for Aboriginal and Torres Strait Islander Children and Youths: A Systematic Review of Data Quality and Population Prevalence
Source: Int J Environ Res Public Health. 2023 Jun 26;20(13):6228. doi: 10.3390/ijerph20136228 (PMC10341665; doi:10.3390/ijerph20136228)
Supplement: Supplementary file 1 [file ijerph-20-06228-s001.zip › ijerph-2413906-supplementary.pdf]

Supplementary Table S1. Prevalence estimates of cardiometabolic risk markers among Indigenous children and youths, arranged by sample cohort type: nationally-representative cohort (dark grey); state or community-representative cohort (light grey); or substantial and longitudinal cohort (white).

**Study details**				**Outcome prevalence**						
**Study, author, year published**	**Study period, setting**	**Sample size, age**	**Study design**	**Increased body mass (obesity)**	**Elevated WC**	**Elevated BP**	**Elevated TG; reduced HDL-C**	**Hyperglycaemia or T2DM**	**Metabolic syndrome**	**JBI[1] score (n/9), CREATE[2] score (n/14), quality issues**
National Aboriginal & Torres Strait Islander Health Survey (NATSIHS)<br><br>Australian Bureau of Statistics, 2019	2018–2019<br><br>Australia-wide	4620<br><br>2–24 years	Cross-sectional study<br><br>Population based	*Pre-school*: 2–3 years 10.8%[3]<br><br>*Children*: 4–8 years 11.5%[3], 9–11 years 13.8%[3]<br><br>*Youths*: 15–17 years 18.3%[3], 18–24 years 32.1%[4]	*Youths*: 18–24 years overall 50.8%[5], males 39.9%, females 62.6%	*Youths*: 18–24 years overall 8.8%[6], males 9.5%, females 7.3%	N/A	N/A	N/A	JBI score 6<br>CREATE score: 10<br>Non-response rates 53.6% for 2–17-year-olds, 34.0% for 18–24-year-olds. Elevated BP diagnosed from one-off measurement.
Australian Aboriginal & Torres Strait Islander Health Survey (AATSIHS)<br><br>Australian Bureau of Statistics, 2013	2012–2013<br><br>Australia-wide	4763<br><br>2–24 years	Cross-sectional study<br><br>Population based	*Pre-school*: 2–4 years 6%[3]<br><br>*Children*: 5–9 years 11.2%[3], 10–14 years 11.8%[3]<br><br>*Youths*: 15–17 years 14.2%[3], 18–24 years 28.4%[4]	*Youths*: 18–24 years males 39.6%[5], females 67.2%	*Youths*: 18–24 years males 8.5%[6], female 4.8%[6]	*Youths*: 18–24 years elevated TG 16.4%[7], reduced HDL-C 29.5%[7]	*Youths*: 18–24 years prediabetes 2.4%[8], diabetes (FBG[9]) 0.1%[7], diabetes (HbA1c[10]) 0.4%[8]	N/A	JBI score: 6<br>CREATE score: 6<br>Underpowered for 18–24-year-olds for biomedical measures (40.4% participation rate). Elevated BP diagnosed from one-off measurement.
O'Dea et al., 2014	2006 and 2012<br><br>Australia-wide	2006: 485<br>2012: 649<br><br>6–18 years	Cross-sectional study<br><br>School based	*Children*: 6–12 years<br>2006 6.7%[3], 2012 5.4%[3]<br><br>*Youths:* 13–18 years 2006 6.5%[3],<br>2012 7.0%[3]	N/A	N/A	N/A	N/A	N/A	JBI score: 8<br>CREATE score: 1<br>Only accounts for children at school. Indigenous children comprised 5.4–5.7% of overall.
Haynes et al., 2016	1990–2012<br><br>WA-wide	76 incident cases<br><br>0–17 years	Retrospective population-based cohort study<br><br>Clinic based	N/A	N/A	N/A	N/A	*Pre-school–youths:* 0–18 years T2DM mean incidence 12.6/100,000 person-years[7]		JBI score: 7<br>CREATE score: 0<br>Ascertainment rate 99% for diagnostic cases. Does not take into account asymptomatic or

Study details				Outcome prevalence						
**Study, author, year published**	**Study period, setting**	**Sample size, age**	**Study design**	**Increased body mass (obesity)**	**Elevated WC**	**Elevated BP**	**Elevated TG; reduced HDL-C**	**Hyperglycaemia or T2DM**	**Metabolic syndrome**	**JBI[1] score (n/9), CREATE[2] score (n/14), quality issues**
										undiagnosed cases.
Tran et al., 2014<br><br>Craig et al., 2007	2001–2008<br><br>NSW-wide	31 incident cases<br><br>0–18 years	Prospective population-based incidence study<br><br>Clinic based	N/A	N/A	N/A	N/A	*Pre-school–youths:* 0–18 years T2DM mean annual incidence 3.0/100,000 per year[8]	N/A	JBI score: 7<br>CREATE score: 0<br>Ascertainment rate 99% for diagnostic cases. Does not take into account asymptomatic or undiagnosed cases.
Wang et al., 2013	1992–2006<br><br>NT, remote Aboriginal community	541<br><br>15–24 years	Matched pair study over two periods (1992–1997, 2004–2006)<br><br>Population based	*Youths:* 15–24 years<br>1992–1997: overall 5.2%[4], males 3.8%, females 7.1%<br><br>2004–2006: overall 5.2%[4], males 4.5%, females 6.3%	N/A	*Youths:* 15–24 years<br>1992–1997: males 11.5%[6], females 7.0%[6]<br><br>2004–2006: males 3.6%[6], females 0.9%[6]	N/A	*Youths:* 15–24 years<br>T2DM 1992–1997: males 1.9%[8], females 1.8%[8]<br><br>2004–2006: males 0.6%[8], female 2.7%[8]	N/A	JBI score: 6<br>CREATE score: 1<br>Coverage of entire community 85%. Elevated BP diagnosed from one-off measurement.
Webster et al., 2013	2005–2007<br><br>NSW, urban western Sydney	122<br><br>24 months	Longitudinal cohort study<br><br>Clinic based	*Pre-school*: 24 months<br>22.1%[11]	N/A	N/A	N/A	N/A	N/A	JBI score: 6<br>CREATE score: 14<br>Small sample size. Coverage of LGA of Campbelltown not reported.
Wolfenden et al., 2011	2007<br><br>NSW, pre-school and day-care centres	78<br><br>2–5 years	Cross-sectional study<br><br>School based, Hunter New England region	*Pre-school:* 2–5 years<br>5.8%[3]	N/A	N/A	N/A	N/A	N/A	JBI score: 6<br>CREATE score: 0<br>Indigenous children comprised 11% of cohort. Response rate of child-care services 59%. 66% of children attending these participated.

Study details				Outcome prevalence						
**Study, author, year published**	**Study period, setting**	**Sample size, age**	**Study design**	**Increased body mass (obesity)**	**Elevated WC**	**Elevated BP**	**Elevated TG; reduced HDL-C**	**Hyperglycaemia or T2DM**	**Metabolic syndrome**	**JBI[1] score (n/9), CREATE[2] score (n/14), quality issues**
Hickie et al., 2013	2004–2008<br><br>ACT, urban and rural kindergartens	321<br><br>4–7 years	Cross-sectional study<br><br>School based	*Pre-school–children:* 4–7 years<br>7%[3]	N/A	N/A	N/A	N/A	N/A	JBI score: 7<br>CREATE score: 3<br>Indigenous participants comprise 1.87% of cohort (compared with 2.5% of national population). Potential sampling bias due to volunteer sampling.
New South Wales Physical Activity and Nutrition Survey (SPANS)<br><br>Hardy et al., 2019<br>Hardy et al., 2014	1997, 2004, 2010, 2015<br><br>NSW, urban and rural primary and secondary schools	1997: 142<br>2004: 123<br>2010: 254<br>2015: 251<br><br>5–16 years	Cross-sectional study<br><br>School based	*Children*: 5–16 years<br>1997: overweight/ obese 23.7%[3]<br><br>2004: overweight/ obese 25.2%[3]<br><br>2010: obese 8.8%[3]<br><br>2015: obese 10.4%[3]	N/A	N/A	N/A	N/A	N/A	JBI score: 6<br>CREATE score: 3<br>Does not include students from remote schools or school with <180 students. Indigenous children comprised 2–3% of cohort. Response rates 57–87% between 1997–2015.
Valery et al., 2009	2003<br><br>Qld, Torres Strait Island school	158<br><br>5–17 years	Cross-sectional study<br><br>School based, Mabuiag, Sue and Thursday Islands	*Children:* 5–17 years<br>15%[3]	*Children:* 5–17 years overall 38%[12], male 13% female 59%	*Children:* 5–17 years<br>27%[12]	*Children:* 5–17 years<br>elevated TG 7%[12] (cohort with normal BMI), 20%[12] (cohort with overweight/obese), reduced HDL-C 41%[12] (cohort with normal BMI, 63%[12] (cohort with overweight/obese)	*Children*: 5–17 years pre-diabetes 8%[12] (cohort with normal BMI), 12%[12] (cohort with overweight/obese)	*Children:* 5–17 years<br>17%[12]	JBI score: 7<br>CREATE score: 11<br>Response rate 98%. Small sample size for biomedical measurements (61.4% of cohort). Elevated BP diagnosed from one-off measurement. Diagnostic cut-off points for 12-year-olds applied to children <12 years.

Study details				Outcome prevalence						
**Study, author, year published**	**Study period, setting**	**Sample size, age**	**Study design**	**Increased body mass (obesity)**	**Elevated WC**	**Elevated BP**	**Elevated TG; reduced HDL-C**	**Hyperglycaemia or T2DM**	**Metabolic syndrome**	**JBI[1] score (n/9), CREATE[2] score (n/14), quality issues**
Longitudinal Study of Indigenous Children (LSIC)<br><br>Thurber, 2013<br>Thurber et al., 2017<br>Deacon-Crouch et al., 2018	2008–2014<br><br>Australia-wide, except Tasmania and ACT	Thurber et al. (waves 1–6) 592–1197, mean age 0.5–9.5 years<br><br>Deacon-Crouch et al. 1124, 6–12 years	Longitudinal cohort study<br><br>Clinic based	*Pre-school–children:*<br>Thurber et al. Younger cohort<br>0.5–1.5 years 13.6%, 1.5–2.5 years 6.7%, 2.5–3.5 years 3.2%, 3.5–4.5 years 3.6%, 4.5–5.5 years 6.3%, 5.5–6.5 years, 13.5%[3]<br><br>Older cohort<br>3.4–4.5 years 7.8%,<br>4.5–5.5 years 8.1%,<br>5.5–6.5 years 9.1%;<br>6.5–7.5 years 11.7%, 7.5–8.5 years 15.0%,<br>8.5–9.5 years 18.2%[3]<br><br>Deacon-Crouch et al.<br>6–12 years 18.5%[3]	N/A	N/A	N/A	N/A	N/A	JBI score: 8<br>CREATE score: 14<br>Recruitment heavily influenced by communities' interest in participating. Not representative for all of Australia but reflective of distribution of Indigenous children across Australia (excepting Tasmania and ACT). Children living in urban centres/major cities underrepresented.
Aboriginal Birth Cohort (ABC)<br><br>Juonala et al., 2016<br>Sjoholm et al., 2020<br>Sellers et al., 2008	1998–2014<br><br>NT, Darwin	Juonala et al. 313–315, 9–20 years<br><br>Sjoholm et al. 315–572, 9–20 years<br><br>Sellers et al. 486, 9–14 years	Longitudinal cohort study<br><br>Clinic based	*Children:*<br>Sjoholm et al. 9–13 years 2.9%[3]<br>Sellers et al. 9–14 years 4.9%[12]<br><br>*Youths*:<br>Sjoholm et al. 16–20 years 6.4%[3]	*Children*: 9–14 years 26.3%[12]	*Children:* 9–14 years (cohort with normal weight but elevated WC) elevated BP 40%[12]	*Children*: 9–14 years (cohort with normal weight but elevated WC) elevated TG 39%[12], reduced HDL-C: 31%[12]	*Children*: 9–14 years (cohort with normal weight but elevated WC) 2%[12]	*Children:*<br>Sellers et al. 9–13 years 15.7%[12]<br><br>Juonala et al. 9–14 years 14%[13]<br><br>*Youths* 16–20 years 16.2%[13]	JBI score: 5<br>CREATE score: 8<br>Only maternal Indigeneity reported. Participants not randomly selected. Elevated BP diagnosed from one-off measurement.

Study details				Outcome prevalence						
**Study, author, year published**	**Study period, setting**	**Sample size, age**	**Study design**	**Increased body mass (obesity)**	**Elevated WC**	**Elevated BP**	**Elevated TG; reduced HDL-C**	**Hyperglycaemia or T2DM**	**Metabolic syndrome**	**JBI[1] score (n/9), CREATE[2] score (n/14), quality issues**
Antecedents of Renal Disease Aboriginal Cohort (ARDAC)<br><br>Kim et al., 2017	2002–2010<br><br>NSW, urban, rural and remote public primary schools	1949–344<br><br>Mean age 10.5–18.5 years	Longitudinal cohort study<br><br>School based	*Children:*<br>Mean age 10.5 years 16.3%[11]<br>12.5 years 19.1%[11]<br>14.5 years 18.6%[11]<br><br>*Youths:* 16.5 years 21.5%[11]<br>18.5 years 19.2%[11]	N/A	*Children:*<br>Mean age 10.5 years 7.3%[14]<br>12.5 years 9.2%[14]<br>14.5 years 9.1%[14]<br><br>*Youths:* 16.5 years 11.1%[14]<br>18.5 years 18.3%[14]	N/A	N/A	N/A	JBI score: 5<br>CREATE score: 10<br>Only included public schools in sampling framework.<br>82.3% of baseline cohort lost to follow-up: 82.3% at 8 years.<br>Elevated BP diagnosed from one-off measurement.
The Study on Environment on Aboriginal Resilience and Child Health (SEARCH)<br><br>Larkins et al., 2017	2008–2011<br><br>NSW, urban<br><br>Aboriginal Community Controlled Health Services	657<br><br>0–17 years	Longitudinal cohort study<br><br>Health service based	*Pre-school–children:* 0–17 years<br>14.8%[4]	N/A	*Pre-school–children:*<br>2–7 years male 16%[14], female 16%[14]<br>8–12 years male 18%[14], female 14%[14]<br><br>*Youths:* 13–17 years male 18%[14], female 11%[14]	N/A	N/A	N/A	JBI score: 5<br>CREATE score: 13<br>Study limited to 4 Aboriginal Community Controlled Health Services in urban and large regional centres in NSW. Elevated BP diagnosed from one-off measurement.<br>64.2% of cohort underwent BP measurement.

[1] Johanna Brigg's Institute
[2] The Centre of Research Excellence in Aboriginal Chronic Disease Knowledge Translation and Exchange
[3] International Obesity Task Force, obesity and overweight defined by age- and sex-specific BMI z-score cut-offs
[4] World Health Organisation, BMI >30kg/m^2
[5] World Health Organisation, WC ≥94cm in males and ≥80cm in females
[6] SBP/DBP ≥140/90mmHg
[7] TG ≥2.0mmol/L; HDL-C <1.0mmol/L males, <1.3mmol/L females
[8] American Diabetes Association, diabetes defined as a) fasting blood glucose ≥7.0mmol/L (≥5.6mmol/L in prediabetes); b) 2-hour oral glucose tolerance test ≥11.1mmol/L (≥7.8mmol/L in prediabetes); c) HbA1c ≥6.5% (between 5.7–6.4% in prediabetes); d) in a patient with clinical symptoms of hyperglycaemia or hyperglycaemic crisis, a random blood glucose ≥11.1mmol/L.
[9] Fasting blood glucose
[10] Glycated haemoglobin
[11] Centres for Disease Control and Prevention (CDC) growth charts, obesity defined by age- and sex-specific BMI z-score cut-offs
[12] National Cholesterol Education Program Adult Treatment Panel III (NCEP ATP III), as outlined in Figure 1
[13] Modified NCEP (National Cholesterol Education Program) (MetSNCEP75), as outline in Figure 1
[14] Fourth Report on the Diagnosis, Evaluation, and Treatment of High Blood Pressure in Children and Adolescent, elevated BP defined by age- and sex-specific z-score cut-offs
